# Evaluation of the effect of a single dose of morroniside on rat liver subjected to ischemia and reperfusion

**DOI:** 10.3389/fmolb.2026.1798488

**Published:** 2026-05-18

**Authors:** Małgorzata Trocha, Aleksandra Kuzan, Paulina Nowotarska, Tomasz Piasecki, Anna Merwid-Ląd, Adam Szeląg, Marcin Magdziarz, Mirosław Sopel, Alicja Z. Kucharska, Katarzyna Madziarska, Tomasz Sozański

**Affiliations:** 1 Clinical Department of Diabetology, Faculty of Medicine, Hypertension and Internal Disease, Wroclaw Medical University, Wroclaw, Poland; 2 Department of Preclinical Sciences, Pharmacology and Medical Diagnostics, Wroclaw University of Science and Technology, Wroclaw, Poland; 3 Department of Biostructure and Animal Physiology, Wroclaw University of Environmental and Life Sciences, Wroclaw, Poland; 4 Department of Epizootiology with Exotic Animal and Bird Clinic, Wroclaw University of Environmental and Life Sciences, Wroclaw, Poland; 5 Department of Pharmacology, Faculty of Medicine, Wroclaw Medical University, Wroclaw, Poland; 6 Hugo Steinhaus Center, Faculty of Pure and Applied Mathematics, Wrocław University of Science and Technology, Wroclaw, Poland; 7 Department of Fruit, Vegetable and Plant Nutraceutical Technology, Wrocław University of Environmental and Life Sciences, Wroclaw, Poland

**Keywords:** hepatic ischemia-reperfusion injury, hepatoprotection, inflammation, morroniside, oxidative stress

## Abstract

**Introduction:**

Hepatic ischemia-reperfusion (IR) injury remains a major clinical problem during liver surgery and transplantation, primarily due to oxidative stress, inflammatory activation, and hepatocellular damage. Morroniside (MO), an iridoid glycoside derived from *Cornus officinalis*, has demonstrated anti-inflammatory, antioxidant, and antifibrotic properties in various experimental models, but its role in hepatic IR injury has not been established. This study aimed to evaluate the effect of a single dose of MO on rat liver subjected to partial ischemia and reperfusion.

**Methods:**

Wistar rats were divided into four groups: control (C), ischemia-reperfusion without treatment (CIR), and IR treated with MO at 90 mg/kg (90MIR) or 270 mg/kg (270MIR). Hepatic injury was assessed biochemically (ALT, AST, PIIINP, antioxidant capacity, cytokine profile), histologically and immunohistochemically (Collagen III, Survivin, HIF-1α, Caspase-8, IL-6).

**Results:**

IR induced significant hepatocellular injury, reflected by elevated aminotransferases, structural disruption, and increased pro-inflammatory cytokines. MO administration produced dose-dependent and heterogeneous effects. At 90 mg/kg, MO partially attenuated early aminotransferase elevation and reduced TNF-α and VEGF levels, suggesting initial hepatoprotective activity; however, hepatocellular damage and fibrosis markers remained elevated after 24 h. At 270 mg/kg, MO paradoxically decreased antioxidant capacity and did not suppress fibrosis or apoptosis, while only modestly reducing pro-inflammatory cytokines. Histopathological analyses confirmed pronounced liver injury in all IR groups regardless of treatment.

**Discussion:**

In conclusion, acute pre-procedural administration of MO did not provide consistent protection against hepatic IR injury, and its effects were dose-dependent and ambiguous. Further studies with prolonged dosing regimens and extended reperfusion times are warranted to clarify MO’s hepatoprotective potential.

## 1 Introduction

Hepatic ischemia-reperfusion (IR) injury represents a significant clinical challenge, occurring, for instance, during liver transplantation or hepatectomy, leading to hepatocyte damage and potential organ failure. The IR process encompasses two phases: ischemia, characterized by oxygen and ATP depletion, resulting in mitochondrial dysfunction and accumulation of reactive oxygen species (ROS), and reperfusion, during which restored blood flow exacerbates oxidative stress, activates Kupffer cells, promotes the production of pro-inflammatory cytokines (such as tumor necrosis factor alpha (TNF-α) and interleukin 6 (IL-6)), and induces apoptosis or necrosis of hepatic cells (Konishi and Lentsch, 2017). These mechanisms also involve the activation of signaling pathways, such as Nuclear Factor kappaB (NF-κB) and Mitogen-Activated Protein Kinase (MAPK), which contribute to inflammation and fibrosis, ultimately leading to chronic liver damage (Teoh, 2011). It is also noted that enhanced lipid peroxidation of cell membranes plays a significant role, leading to apoptosis or necrosis, while the nuclear protein High Mobility Group Box 1 (HMGB1) exerts a protective role, contributing to tissue repair and regeneration (George et al., 2024). The specificity and complexity of hepatic IR injury make it an intriguing experimental model for evaluating the protective effects of various compounds.

Morroniside (MO), an iridoid glycoside derived from the fruits of *Cornus officinalis*, a plant traditionally used in Chinese medicine (Ha et al., 2012), exhibits beneficial effects on lipid metabolism and possesses anticancer, antiseptic, and antidiabetic properties (Jiang et al., 2011; Jiang W. et al., 2025; Chen et al., 2014; Wang et al., 2025). It also inhibits the expression of pro-inflammatory cytokines and adhesion molecules, exerts anti-apoptotic and antioxidant effects by increasing superoxide dismutase (SOD) and catalase activity, reducing reactive oxygen species (ROS) levels, and activating the Nuclear factor erythroid 2-related factor 2 (Nrf2) and Heme Oxygenase-1 (HO-1) (Nrf2/HO-1) pathway (Shi et al., 2024). By promoting angiogenesis and improving blood flow, it demonstrates cardioprotective (Jiang et al., 2011; Liu et al., 2020) and neuroprotective effects (Wang et al., 2010). Existing studies also suggest its hepatoprotective potential, although most research has utilized other experimental models (Park et al., 2010). In non-alcoholic steatohepatitis (metabolic-associated steatohepatitis, MASH), MO inhibits NLRP3 inflammasome activation, promotes AMPK-dependent lipophagy, and modulates lipid metabolism, thereby reducing inflammation and fibrosis (Zhang et al., 2024).

Liver transplantation remains the only effective treatment for end-stage liver failure. Given the persistent shortage of donor organs and high clinical demand, the identification of novel hepatoprotective agents against ischemia-reperfusion (IR) injury is of major importance. Although many compounds show protective effects when administered chronically, agents capable of providing benefit after a single pre-procedural dose are particularly attractive for clinical use. Such acute preconditioning could be especially valuable in urgent liver surgery and transplantation, where prolonged pretreatment is often not feasible.

Morroniside, an iridoid glycoside derived from *C. officinalis*, exhibits multifaceted protective properties, including antioxidant, anti-inflammatory, and organ-protective effects in various experimental models. To our knowledge, this study represents the first evaluation of morroniside administered as a single acute dose in a model of hepatic IR injury.

## 2 Materials and methods

### 2.1 Animals

The study was conducted on male Wistar rats, aged between 10 and 12 weeks. The rats were kept in separate chambers under standard conditions, including a 12-h light/12-h dark cycle, humidity levels of 45%–60%, constant ventilation, and a temperature range of 21 °C–23 °C.

### 2.2 Ethical approval and informed consent

All procedures performed in the study were in accordance with the ethical standards of the institutional and/or national research committee. All applicable international, national, and/or institutional guidelines for the care and use of animals were followed. The experiment protocol was approved by the Local Ethics Committee on the Animal Research of the Institute of Immunology and Experimental Therapy, Polish Academy of Sciences in Wroclaw (040/2020/P1 of 21 October 2020).

### 2.3 Chemicals

Morroniside (CAS No. 25406-64-8, purity >98% as certified by the supplier via HPLC) was obtained from ChemNorm (Wuhan, China). No additional independent purity verification was performed in the present study. However, the batch was used directly according to the manufacturer’s certificate of analysis. For anesthesia during the isolation and ligation of the hepatic artery and portal vein branches and for euthanasia, Isoflurane (Isotek, 1,000 mg/g solution, 250 mL, Vet-Argo Trading, Lublin, Poland) was administered via inhalation at 4% concentration for induction and 2% for maintenance. Butorphanol tartrate (Morphasol, 4 mg/mL ampoules, aniMedica GmbH, Frankfurt, Germany) was employed for anesthesia and analgesia during ischemia, post-reperfusion recovery, and euthanasia, administered intramuscularly at a dose of 2 mg/kg body weight. To extend analgesic effects during the ischemia-reperfusion (IR) procedure, Metamizole sodium (Pyralgivet, 500 mg/mL solution, 100 mL, Vet-Agro, Lublin, Poland) was used. Heparin (Heparinum WZF, 25,000 U/5 mL ampoules, Polfa Warszawa, Poland) was applied to prevent blood clotting. To restore vascular volume, 0.9% sodium chloride solution (Polpharma S.A., Starogard Gdański, Poland) and Ringer solution (Polfa Lublin S.A., Poland) were utilized in the experiment.

### 2.4 Experimental design

Following a 14-day adaptation period, rats were randomly assigned to four groups. In groups C (n = 12) and CIR (n = 12), animals were not treated with MO. In contrast, groups 90MIR (n = 12) and 270MIR (n = 12) received MO orally at doses of 90 mg/kg and 270 mg/kg, respectively, as a single dose the day prior to surgery. The livers of groups CIR and MIR underwent the IR procedure. Blood samples were collected from the tail vein after MO administration to measure baseline alanine aminotransferase (ALT) and aspartate aminotransferase (AST) activity.

The study limited the number of animals to the minimum required, determined by the reliability of statistical analysis. Based on an assumed average population value for one monitored parameter in Wistar rats aged 10–12 weeks at 24 h of reperfusion (0.73 ± 0.17 μmol/L), a minimum expected difference of 0.21, a Type I error probability (α) of 0.05, and a target test power (β) of 0.8, the required sample size was calculated as 12. Sample size estimation was based on ADMA levels as the primary endpoint, using variance derived from prior experiments in the same model. The study was not powered for all secondary endpoints individually; therefore, findings related to secondary outcomes should be considered exploratory. The calculations were performed using STATISTICA Software (version 13.3, StatSoft, Inc.). The MO dosage and oral administration route were determined based on prior animal studies (Wang et al., 2010).

### 2.5 IR procedure

Rats were anesthetized using inhaled isoflurane and intramuscular butorphanol (2 mg/kg). Under general anesthesia, a midline laparotomy was performed. In groups CIR and MIR, the hepatic artery and portal vein branches were clamped with a microvascular clip, inducing ischemia in 70% of the liver (median and left lateral lobes). Heparin (200 U/kg) was administered to prevent blood clotting. After 60 min of ischemia, the clip was removed to initiate 24 h of reperfusion. Blood samples were taken at 3 and 24 h of reperfusion to assess aminotransferase activity. Upon experiment completion, livers were weighed, ischemic lobes were isolated, and a portion of the lobes was preserved in 10% formalin for further analyses (e.g., immunohistochemistry (IHC)).

In group C, rats underwent identical anesthesia and surgical procedures as the ischemic groups (CIR, MIR), but the hepatic artery and portal vein branches were not clamped following midline laparotomy. Blood samples were collected at the same time points as in the ischemic groups. After 24 h of reperfusion, the same liver lobes were isolated as in the ischemic groups. All surgical procedures, whether involving IR or not, were conducted blindly by the same experienced research team.

### 2.6 Biochemical analyses

Serum concentrations of the N-terminal propeptide of type III procollagen (PIIINP) were determined using an ELISA kit (*Rat PIIINP/N-terminal propeptide of Collagen alpha-1(III) chain ELISA Kit*, EIAab Science Co., Ltd., Wuhan, China; Cat. No. E0573r). Antioxidant capacity was measured in the same serum samples with the *Antioxidant Assay Kit* (Cayman Chemical, Ann Arbor, MI, United States; Cat. No. 709001-96). All assays were performed according to the manufacturers’ protocols.

Cytokine concentrations in rat serum samples were quantified using a bead-based multiplex immunoassay (*ProcartaPlex Rat Expanded Cytokine Magnetic Bead Panel*, Thermo Fisher Scientific, Waltham, MA, United States; Cat. No. RECYTMAG-65K-09), according to the manufacturer’s instructions and analyzed on a Luminex® platform (Thermo Fisher Scientific).

The levels of aminotransferases (ALT, AST) in serum were measured using a commercially available enzymatic method at a certified laboratory.

### 2.7 Histological examination

Various regions of the isolated liver lobes from both ischemic and non-ischemic groups were preserved in 10% formalin and embedded in paraffin. Sections measuring 4.5 μm were prepared and stained with hematoxylin-eosin. These sections sections were scanned and analyzed using the Olympus Slideview VS200 digital slide scanner (Olympus Corporation, Tokyo, Japan) to assess the extent of ischemic necrosis, the degree of steatosis (expressed as a percentage of the microscopic field, characterized by small cytoplasmic vacuoles containing lipids or single fat droplets displacing hepatocyte nuclei), neutrophil infiltration, and disruption of hepatic architecture.

### 2.8 Immunohistochemistry (IHC)

Immunohistochemical staining was performed on liver tissue sections using the *ImmPRESS®-HRP Universal (Horse Anti-Mouse/Rabbit IgG) PLUS Polymer Kit, Peroxidase* (Vector Laboratories, Burlingame, CA, United States; Cat. No. MP-7800-15), according to the manufacturer’s protocol. The following primary antibodies were applied: Survivin (Affinity Biosciences, Cat. No. AF6017), IL-6 (Affinity Biosciences, Cat. No. DF6087), HIF-1α (Affinity Biosciences, Cat. No. AF1009), Collagen type III (Mouse monoclonal antibody, Affinity Biosciences, Cat. No. BF9208), and Caspase-8 (Affinity Biosciences, Cat. No. AF6442). Detection was carried out using the ImmPRESS®-HRP kit with diaminobenzidine (DAB) as chromogen, and hematoxylin was used for nuclear counterstaining. Negative controls were prepared by omitting the primary antibody. Immunostained sections were scanned and analyzed using the *Olympus Slideview* VS*200 digital slide scanner* (Olympus Corporation, Tokyo, Japan). Analysis was performed semi-quantitatively using a staining intensity scale from 0 to 3 (0 = no reaction, 3 = very strong reaction), and the percentage of cells showing a positive reaction in the visual fields was determined. Each slide was evaluated independently in several representative areas, including the periportal and pericentral zones. All immunohistochemical evaluations were performed by two independent observers blinded to group allocation.

### 2.9 Statistical analysis

Statistical methods appropriate to the type and distribution of variables were employed for data analysis. Initially, the normality of data distribution was assessed using the Shapiro-Wilk test. For data exhibiting a normal distribution, analysis of variance (one-way ANOVA) was conducted to compare means between groups. In cases where significant differences were identified, *post hoc* tests (Tukey’s test) were applied to determine specific group differences. For data not meeting normality assumptions, the non-parametric Kruskal–Wallis test was used, followed by Dunn’s test for *post hoc* analysis.

For dependent samples (ALT and AST measurements at different time points), repeated-measures ANOVA was utilized for normally distributed data, while the Friedman test was applied for non-normal data.

Means, standard deviations, medians, and interquartile ranges were calculated. To enhance data visualization, results were presented as bar plots. Additionally, correlation analysis between selected biological parameters was performed using Pearson’s correlation coefficient. A statistical significance level of α = 0.05 was adopted. The analyses were performed using Statistica (13.3, TIBCO Software Inc., United States) and Matlab (R2024b, MathWorks, United States). Figures were prepared using the GraphPad Prism version 8.0 (GraphPad Software, San Diego, CA, United States).

## 3 Results

### 3.1 Blood tests

**FIGURE 1:**
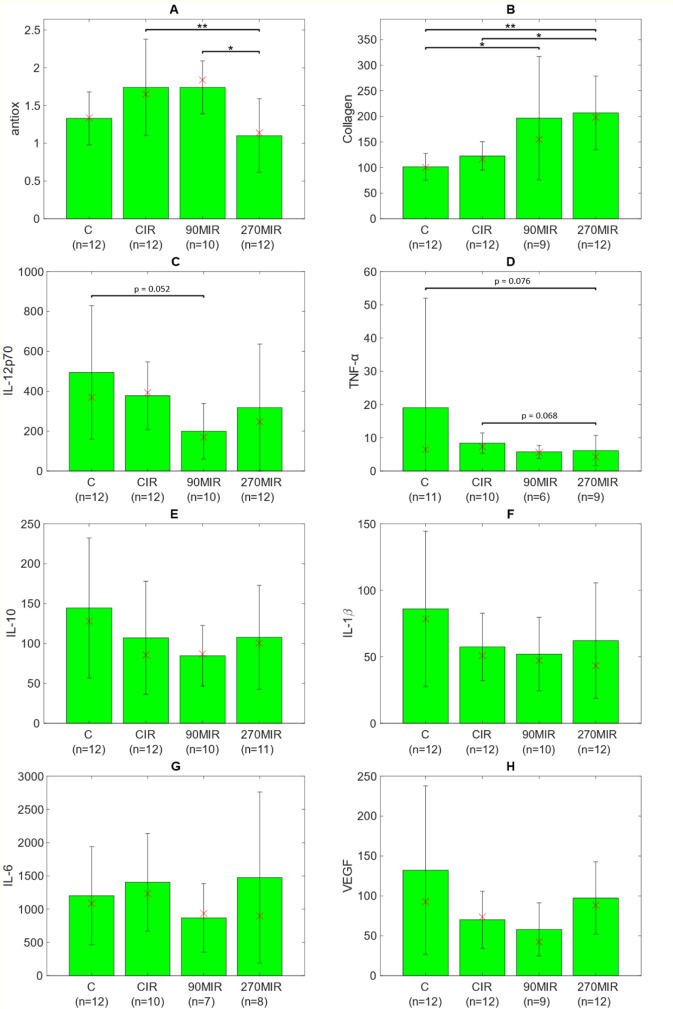
Bar plots showing means and standard deviations of the blood parameters for each group. Panel **(A)**—antioxidant capacity; Panel **(B)**—collagen content; Panel **(C)**—IL-12p70 concentration; Panel **(D)**—TNF-α concentration; Panel **(E)**—IL-10 concentration; Panel **(F)**—IL-1β concentration; Panel **(G)**—IL-6 concentration; Panel **(H)**—VEGF concentration. Additionally, medians are marked with ‘x’ and sample sizes are displayed below group labels. The asterisks above the brackets indicate the p-value level of the statistical test ( * - p < 0.05, ** - p < 0.01). P-values very close to the significance level are also given.

Regarding oxidative stress parameters, a significant difference was observed, with the lowest levels in the 270MIR group compared to the CIR and 90MIR groups (p = 0.004) (Figure 1A).

Significantly higher collagen levels were found in the 90MIR, and 270MIR groups compared to the control group (C), as well as in the 270MIR group compared to the CIR group (p = 0.0008) (Figure 1B). A strong correlation was identified between collagen and ALT3/AST3 (ρ up to 0.84) across all intervention groups.

Statistically significant differences were observed in IL-12p70 cytokine levels among the studied groups (p = 0.0291). Post-hoc analysis revealed that the differences in IL-12p70 levels approached statistical significance between the C and 90MIR groups (p = 0.052) (Figure 1C). Significant differences were observed in TNF-α levels (p = 0.0342) among the studied groups. Post-hoc statistical analysis showed that the largest differences were observed between the C and 270MIR groups (p = 0.076), and the CIR and 270MIR groups (p = 0.068), approaching statistical significance (Figure 1D). Differences in levels of other (IL-10, IL-1β, IL-6) cytokines were not statistically significant (Figures 1E–G). However, very strong correlations were observed in the C and CIR groups between IL-1β, IL-10, and TNF-α, and in the 270MIR group between IL-6 and TNF-α (ρ ≈ 0.99).

No significant differences were observed for VEGF levels (p = 0.098), although the lowest values were noted in the 90MIR group (Figure 1H). VEGF was strongly correlated with IL-10 and TNF-α in the C and CIR groups.

### 3.2 Analysis of ALT and AST

Significant differences in ALT and AST levels were observed at 3 h of reperfusion between the groups (p < 0.001 for both comparisons). Post-hoc analysis confirms significant differences between group C and the remaining groups. Mean ALT3 and AST3 levels significantly increased in the IR groups, with the highest values observed in the CIR group and the lowest among the IR groups in the 90MIR group. At 24 h of reperfusion, significant differences in ALT and AST levels were still observed between the groups (p < 0.001 for both comparisons). These results were confirmed in a *post hoc* analysis. Interestingly, in the 90MIR group, the highest mean and median values for ALT24 and AST24 were observed, distinguishing it from other IR groups, where ALT24 and AST24 values decreased compared to ALT3 and AST3, respectively.

**FIGURE 2:**
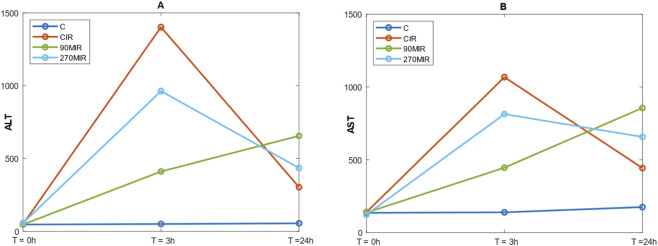
Plots showing medians of ALT (panel **(A)**) and AST (panel **(B)**) changing in time, for each of the analyzed groups.

Regarding changes in aminotransferase activity over time, statistically significant differences in aminotransferase levels were observed at baseline, at 3 h, or at 24 h of reperfusion for all groups subjected to IR injury. Post-hoc analysis confirmed significant differences in ALT values across the three measured time points for all I/R groups. Regarding AST, *post hoc* analysis revealed significant differences across all time points for the CIR and 270MIR groups. In the 90MIR group, only the difference between AST0 and AST24 was important (Figures 2A,B; Table 1). Additionally, very strong correlations were observed between ALT3 and AST3, as well as between ALT24 and AST24.

**TABLE 1:** Means, standard deviations and sample sizes of ALT and AST changing in time, for each of the analyzed groups.

ALT			
Time group	T = 0h	T = 3h	T = 24h
C	46,50 ± 7,01 (n = 12)	52,42 ± 8,24 (n = 12)	62,42 ± 28,34 (n = 12)
CIR	49,75 ± 6,93 (n = 12)	1324,90 ± 519,15 (n = 10)	536,17 ± 526,17 (n = 12)
90MIR	53,00 ± 22,58 (n = 10)	842,89 ± 1098,10 (n = 9)	1254,10 ± 1621,19 (n = 10)
270MIR	61,67 ± 22,39 (n = 12)	1113,67 ± 919,09 (n = 12)	721,50 ± 768,70 (n = 12)

AST			
C	151,42 ± 39,00 (n = 12)	164,00 ± 60,54 (n = 12)	235,42 ± 175,38 (n = 12)
CIR	155,08 ± 46,17 (n = 12)	1210,00 ± 619,09 (n = 10)	625,75 ± 445,08 (n = 12)
90MIR	140,40 ± 48,50 (n = 10)	743,89 ± 786,59 (n = 9)	1283,30 ± 1308,66 (n = 10)
270MIR	155,25 ± 69,44 (n = 12)	1172,33 ± 1005,52 (n = 12)	961,92 ± 810,33 (n = 12)

### 3.3 Histopathological evaluation

#### 3.3.1 Histological findings

**FIGURE 3:**
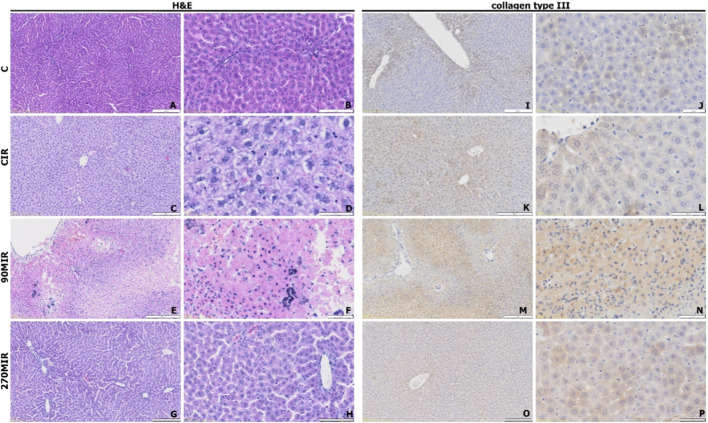
Representative images of liver tissue sections showing histological and immunohistochemical analyses; hematoxylin and eosin (H&E) staining illustrating general tissue morphology (panels **(A–H)**), immunohistochemical staining for collagen type III, demonstrating its distribution and relative abundance within the tissue (panels **(I–P)**).

H&E-stained liver sections revealed distinct morphological differences across study groups. In group C, livers exhibited normal histological structure, with intact hepatic cords, no pathological changes, and erythrocytes in vascular lumina indicating proper perfusion (Figures 3A,B). The CIR group showed severe hepatocellular damage, including shrunken cells and disrupted tissue integrity (Figures 3C,D). In the 90MIR group, hepatocytes were enlarged, irregularly shaped, and less hematoxylin-stained, with numerous cells displaying basophilic, granular cytoplasm, likely activated Kupffer cells, and areas of rarefaction beneath the central vein endothelium (Figures 3E,F). In the 270MIR group, hepatocytes had granular cytoplasm, disrupted hepatic cords, and intense eosin staining, suggestive of necrosis (Figures 3G,H).

#### 3.3.2 Immunohistochemical reactions

##### 3.3.2.1 Collagen III expression

Collagen III was detected in all groups (Figures 3I–P), primarily around central veins, with concentric distribution in the cytoplasm and extracellular matrix. In groups C and CIR (Figures 3I–L), no expression was observed in portal spaces. The 90MIR group showed focally stronger immunohistochemical staining compared to controls (Figures 3M,N), while the 270MIR group exhibited moderate expression, predominantly radial from central veins, with slightly enhanced staining in some areas unrelated to portal space (Figures 3O,P).

##### 3.3.2.2 Survivin expression

**FIGURE 4:**
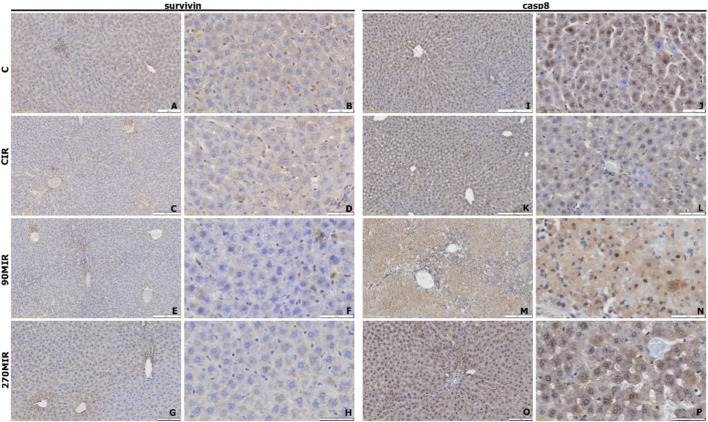
Representative microphotographs of tissue sections immunohistochemically stained for apoptosis-related antigens: survivin (panels **(A–H)**) and caspase-8 (panels **(I–P)**).

In group C, survivin expression was high in nearly all cells, with uniform staining and occasional antigen accumulation along cell membranes (Figures 4A,B). In the CIR group, expression was reduced, mainly limited to vascular endothelial cells, selected hepatocytes near central veins, and cells with macrophage morphology (Figures 4C,D). The 90MIR group showed similarly low expression in a small subset of cells (Figures 4E,F). In the 270MIR group, moderate expression was observed, distributed perivascularly, in portal areas, and scattered within hepatocytes (Figures 4G,H).

##### 3.3.2.3 CASP8 expression

In group C, CASP8 was detected in hepatocyte cytoplasm and nuclei, with no staining in Kupffer or endothelial cells (Figures 4I,J). The CIR group showed a marbled pattern of alternating positive and negative cells, with slightly lower intensity than group C (Figures 4K,L). In the 90MIR group, high-intensity staining was diffuse in cytoplasm and nuclei, with reduced staining near hepatic triads and no concentration around central veins (Figures 4M,N). The 270MIR group showed similar localization but with moderate intensity (Figures 4O,P).

##### 3.3.2.4 HIFα expression

**FIGURE 5:**
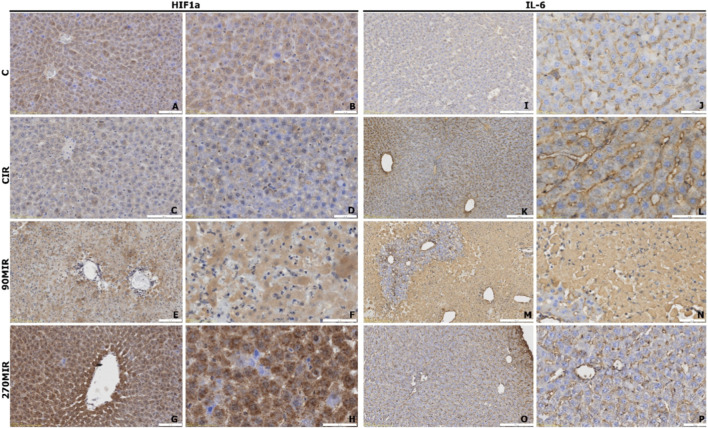
Representative microphotographs of tissue sections immunohistochemically stained for antigens related to the response to stress and inflammation: HIF1α (panels **(A–H)**) and IL-6 (panels **(I–P)**).

In group C, HIFα expression was pronounced, mainly cytoplasmic in hepatocytes, with some nuclear localization; Kupffer cells remained unstained (Figures 5A,B). The CIR group showed reduced cytoplasmic expression compared to group C (Figures 5C,D). The 90MIR group exhibited moderate cytoplasmic expression with slight antigen accumulation around central veins (Figures 5E,F). The 270MIR group displayed the highest expression, predominantly cytoplasmic with granulation, focally overloaded cells, and pericentral antigen concentration, with no staining in Kupffer cells (Figures 5G,H).

##### 3.3.2.5 IL-6 expression

IL-6 was localized in hepatocyte cytoplasm across all groups, with brown granules accumulating along sinusoids (Figures 5I–P). Group C showed moderate expression (Figures Fig5I–J). In the CIR group, staining intensity varied, with weaker staining at the lobular periphery and stronger reactivity near central veins (Figures 5K,L). The 90MIR group lacked staining around hepatic triads, forming ∼50 µm hematoxylin-only concentric regions, though vascular endothelium in triads showed positive immunohistochemical reactivity (Figures 5M,N). The 270MIR group exhibited less intense staining compared to 90MIR (Figures 5O,P).

The expression of the analyzed antigens, defined as the intensity of the immunohistochemical reaction, was assessed semi-quantitatively by direct visual microscopic evaluation. The results are presented as a comprehensive summary of all analyzed samples and are shown in Table 2.

**TABLE 2:** Semi-quantitative assessment of the immunohistochemical reaction.

Analyzed antigen	Control C	Control CIR	90MIR	270MIR
Collagen III	1.25 (40%)	1.75 (65%)	1.9 (50%)	1.75 (45%)
Survivin	1.75 (95%)	1 (7.5%)	1 (15%)	1.5 (20%)
HIF1a	2.25 (92.5%)	1.5 (87.5%)	2 (90%)	2.5 (92.5%)
Casp8	2.5 (91%)	2.15 (80%)	2.3 (85%)	2 (92%)
IL-6	1.5 (90%)	2 (82.5%)	2.5 (80%)	1.25 (75%)

Assessment of the intensity of the immunohistochemical reaction on a three-point scale, where 0 represents no reaction and 3 represents a very intense reaction. The percentages in parentheses represent the proportion of cells exhibiting an immunohistochemical reaction.

Abbreviations: HIF1A, Hypoxia-Inducible Factor 1-alpha; CASP8, Caspase-8; IL-6, Interleukin-6; 90MIR, group subjected to IR, and treated with 90 mg/kg of morroniside, 270MIR: group subjected to IR, and treated with 270 mg/kg of morroniside CIR, untreated group subjected to IR, and C–untreated and sham-operated group.

## 4 Discussion

Available studies suggest that MO, through its antioxidant and anti-inflammatory effects, may exert a protective role in various models of liver injury (Wang et al., 2009). However, IR injury, characterized by acute oxidative stress, activation of inflammatory pathways, mitochondrial damage, and Kupffer cell activation (Jaeschke, 2003), represents a specific form of organ damage. To date, no studies have investigated the effect of MO on hepatic ischemia-reperfusion (IR) injury. However, analyses of IR injury in other organs suggest that it may also have a protective effect in the liver (Wang et al., 2025). It has been demonstrated that other iridoids reduce HMGB1 release and inflammation in the liver subjected to IR (Guo et al., 2025). In the present study, single-dose administration of MO immediately prior to IR produced inconsistent, dose-dependent effects on inflammation, fibrosis, angiogenesis, and antioxidant parameters.

In groups of rats subjected to IR, typical structural changes in the liver were observed, including necrosis, inflammatory infiltrates, and disrupted trabecular architecture. These findings correlated with elevated aminotransferase levels and indicated the induction of acute liver injury, consistent with previous studies (Arab et al., 2009). In the IR group, a sharp increase in aminotransferases was observed at 3 h of reperfusion, with a partial decline after 24 h, aligning with the typical course of reperfusion injury, where initial hepatocyte necrosis leads to enzyme release, followed by a regeneration phase (Trocha et al., 2019). In the MO-treated IR groups, the rise in aminotransferase levels was less pronounced compared to the untreated group, indicating a protective effect of MO during the early reperfusion phase, especially at a lower dose. However, after 24 h of reperfusion, an unexpected further increase in aminotransferases was observed only in the group treated with the low dose of MO, accompanied by pronounced morphological changes such as signs of early necrosis and neutrophil infiltrates (see Figures 3E,F). This pattern suggests delayed hepatocyte damage despite an initial protective effect. This finding is surprising in the context of literature indicating the hepatoprotective effects of MO in various liver injury models. However, it has been suggested that the effects of iridoids on liver and cardiovascular function are dose-dependent, and higher concentrations may induce hepatotoxicity, as observed with another iridoid, geniposide (Danielewski et al., 2020). In the present study, the lack of a clear protective effect may thus result from differences in dosing or the acute dosing regimen used.

Inflammation is a central component of hepatic IR injuryNF-α, a key pro-inflammatory cytokine, is rapidly released by hepatic macrophages during reperfusion, triggering an inflammatory cascade and promoting hepatocyte apoptosis (Perry et al., 2011). While low levels of TNF-α may exert protective preconditioning effects, excessive production exacerbates tissue damage (Teoh, 2011). Reducing this cytokine decreases neutrophil infiltration and lung injury following hepatic IR (Sorkine et al., 1995), and TNF-α inhibitors, such as soluble receptors, prevent remote organ damage after liver transplantation (Welborn MB, 1996). IL-12 also plays a significant role in the pathogenesis of IR injury. It is essential for neutrophil recruitment and amplification of the immune response (Lentsch et al., 1999). Moreover, IL-12-mediated interactions between innate and adaptive immunity significantly aggravate liver damage during reperfusion (Zhai et al., 2011), whereas blockade of IL-12 markedly reduces the severity of IR-induced injury (Konoeda et al., 2010). MO has been shown to inhibit the production of pro-inflammatory cytokines, such as TNF-α and IL-6, by affecting the NF-κB pathway (Chen et al., 2022). By suppressing the levels and expression of pro-inflammatory cytokines, it mitigates experimentally induced autoimmune encephalitis in mice (Jiang T. et al., 2025), improves bone metabolism and quality in diabetic rats (Yang et al., 2025), and reduces joint destruction in rheumatoid arthritis (Wang Y, 2024). To date, the effect of MO on IL-12 levels in hepatic IR injury has not been studied. We only know that other iridoids, by inhibiting IL-12, exert a protective effect in conditions such as psoriasis (Liu et al., 2024) and autoimmune hepatitis (Wang et al., 2023). In our study, MO reduced TNF-α levels at the higher dose, which may suggest suppression of the inflammatory response following IR. This observation is supported by the presence of lower IL-12p70 levels at the lower dose of MO; however, the borderline statistical significance does not allow for firm conclusions to be drawn. Surprisingly, high levels of these cytokines were observed in the control group, which may be attributed to a nonspecific response to micromanipulation. Additionally, in the MO-treated groups, strong correlations were observed between IL-6 and IL-12p70, as well as IL-6 and TNF-α, while in the control and CIR groups, strong inter-cytokine correlations were found between IL-1β, IL-10, and TNF-α. These associations reflect the coordinated pro-inflammatory cytokine network activated by oxidative stress and tissue damage during hepatic IR (Bataller and Brenner, 2005; Kaltenmeier et al., 2022).

Oxidative stress plays a central role in the pathogenesis of hepatic ischemia-reperfusion (IR) injury. During ischemia, oxygen deprivation disrupts mitochondrial function, leading to the accumulation of reactive oxygen species (ROS) (Jaeschke, 2003). Upon reperfusion, excessive ROS production damages cellular membranes, proteins, and DNA, activates inflammatory pathways, and ultimately results in hepatocyte death (Eltzschig and Eckle, 2011). Although previous studies have consistently demonstrated the antioxidant and anti-inflammatory properties of morroniside under chronic or repeated dosing regimens (e.g., via activation of the Nrf2/HO-1 pathway, upregulation of SOD and catalase, and ROS scavenging) (Park et al., 2009; Shi et al., 2024; Wang et al., 2009), the present findings reveal dose-dependent and partially paradoxical effects following acute single-dose administration of MO. Antioxidant activity, assessed as the liver’s capacity to neutralize ROS, was comparable between the low-dose MO group and the ischemic control group. In contrast, significantly lower values were observed in the high-dose MO group compared with both the ischemic control and the low-dose group. This may reflect a biphasic response in which high acute concentrations of iridoids exert pro-oxidant effects through direct interactions with redox-active metal ions or transient overload of cellular detoxification systems, as previously suggested for structurally related compounds such as geniposide (Danielewski et al., 2020).

Wang et al. reported strong antioxidant effects of MO at a dose of 270 mg/kg in a cerebral IR model, where the compound was administered for 7 days prior to IR induction, potentially enabling preconditioning and upregulation of endogenous antioxidant defenses, in contrast to the acute dosing regimen used in our study (Wang et al., 2010). Although the Nrf2/HO-1, NF-κB, and TGF-β/Smad pathways were not directly assessed in our study, the obtained results allow us to speculate that acute exposure to MO does not provide sufficient time for transcriptional activation of the Nrf2/HO-1 pathway or other adaptive antioxidant mechanisms that require sustained stimulation. However, mechanistic validation of these hypotheses requires further targeted molecular analyses.

The strong correlation between antioxidant parameters and aminotransferase levels suggests direct link between the intensity of oxidative stress and the severity of hepatocyte injury (Li et al., 2015). Notably, the absence of consistent hepatoprotective effects, despite a modest early attenuation of aminotransferase release at 90 mg/kg, indicates that the pharmacodynamic profile of morroniside under conditions of acute, severe oxidative stress during hepatic IR differs substantially from that observed in milder, progressive models of liver injury (e.g., MASH or toxin-induced damage (Zhang et al., 2024)). These results highlight the importance of timing and duration of exposure in modulating the compound’s overall biological effect.

In hepatic IR injury, an increase in VEGF promotes angiogenesis but also exacerbates tissue damage (Tamagawa et al., 2008). MO, through its antioxidant and anti-inflammatory effects, may suppress VEGF expression via HIF-1α pathways, similar to the observed effects of resveratrol, where suppression of HIF-1α/VEGF reduced liver damage in IR (Zhang et al., 2014). In our study, VEGF levels were lowest in the group treated with the lower dose of MO, suggesting a potential dose-dependent anti-angiogenic effect. However, statistical significance was observed only in comparison to the control group, which exhibited the highest VEGF levels. These results require further investigation to confirm MO’s protective effects. Additionally, no suppression of HIF-1α expression was observed, as it remained comparably high across all groups.

Liver fibrosis is a complex process involving the activation of hepatic stellate cells (HSCs), which, in response to injury such as ischemia-reperfusion (IR), produce type I and III collagen, leading to extracellular matrix (ECM) remodeling (Bataller and Brenner, 2005). HSC activation occurs via pathways such as TGF-β/Smad under conditions of increased oxidative stress and inflammation (Friedman, 2008). Following IR injury, fibrogenesis may support tissue repair but can also contribute to chronic damage (Konishi et al., 2019). In our study, the observed increase in serum collagen levels and immunohistochemical expression of collagen III within 24 h of reperfusion most likely reflects early extracellular matrix (ECM) remodeling and provisional matrix deposition rather than established fibrosis. Elevated serum procollagen type III N-terminal propeptide (PIIINP) is recognized as a sensitive marker of ongoing ECM turnover and tissue repair processes, rather than progressive fibrogenesis (Hasselbalch et al., 2023). In the context of hepatic IR, such rapid collagen deposition may represent an attempt to stabilize tissue following hepatocyte necrosis and inflammatory activation. The strong positive correlation between collagen III and aminotransferase levels at 24 h further supports the interpretation that early extracellular matrix remodeling in hepatic IR injury is closely associated with the severity of acute hepatocellular injury. IR-induced damage promotes fibrogenesis through activation of inflammatory pathways (Szabo and Petrasek, 2015). Nevertheless, longer observation periods would be required to determine whether this early remodeling progresses toward pathological fibrosis or resolves during regeneration (Nielsen et al., 2015).

Morroniside (MO) has been described in the literature as a compound with potential antifibrotic properties, partly through inhibition of hepatic stellate cell activation (An et al., 2022). In a pulmonary fibrosis model, MO reduced the expression of collagen fibers, hydroxyproline, TGF-β1, collagen I, and α-SMA mRNA in lung tissue (Chen et al., 2022). However, in our study, collagen levels were significantly higher in both MO-treated groups compared with the non-IR group, with no differences relative to the untreated IR group. This suggests that the antifibrotic effects of MO observed in other models of liver injury may not be present in the IR model, particularly under conditions of single-dose administration.

Regarding apoptosis parameters, survivin expression was low in IR groups, while caspase-8 expression was high, suggesting inhibition of apoptosis. This aligns with partial hepatocyte regeneration, as evidenced by a decline in aminotransferase levels at 24 h of reperfusion. Interestingly, survivin inhibition has been shown to attenuate liver fibrosis by inducing HSC senescence and reducing hepatic macrophage populations (Cho et al., 2010; Sharma et al., 2024). MO has demonstrated anti-apoptotic effects in various organ injury models, including brain IR (Zeng et al., 2018). However, in our hepatic ischemia-reperfusion (IR) injury model, no anti-apoptotic effect of MO was observed with a single-dose administration.

In summary, the effect of MO on rat liver subjected to IR, when administered only on the day before the surgical procedure, is ambiguous and dose-dependent. Notable results were observed with the lower dose of 90 mg/kg, which appears to exert a protective effect by inhibiting pro-inflammatory cytokines and VEGF. However, the continued rise in aminotransferases and pronounced histopathological changes in this group suggest that these results require further investigation, particularly with modified dosing regimens involving administration for several days prior to the procedure. The higher dose of 270 mg/kg reduced antioxidant activity, which contradicts the expected antioxidant properties of MO and indicates a paradoxical effect potentially exacerbating oxidative stress. No inhibition of fibrosis or apoptosis was observed.

Our study has certain limitations. First, we used a single-dose administration of morroniside given the day before surgery. While this acute pre-procedural approach constitutes a novel aspect of the study and reflects a clinically relevant scenario (e.g., preconditioning before urgent liver surgery or transplantation), it may also be viewed as a limitation. The observed limited and heterogeneous effects may partly result from insufficient time for cellular accumulation, metabolic activation, or induction of adaptive cytoprotective pathways that typically require sustained exposure. Second, the 24-h reperfusion period, although sufficient to capture acute hepatocellular injury, does not adequately address the time-dependent processes of liver regeneration and fibrotic remodeling. In future studies, it will likely be necessary to consider an extended reperfusion period (e.g., 48–72 h), which would allow for a better assessment of long-term fibrotic changes and regenerative responses. Furthermore, the acute single-dose model combined with the lack of pharmacokinetic data limits the translational relevance of our results. Without information on absorption, distribution, and liver tissue concentrations of morroniside, direct extrapolation to clinical practice remains difficult.

Additional limitations include the absence of direct oxidative stress markers, such as malondialdehyde (MDA) and 4-hydroxynonenal (4-HNE) levels or antioxidant enzyme activities (SOD, catalase), and the lack of analysis of molecular pathways such as Nrf2/HO-1, precludes a definitive assessment of MO’s antioxidant effects in the IR model and thus does not allow confirmation of the lack of efficacy of this compound following a single dose administered immediately prior to the procedure. Additionally, the lack of analysis of molecular mechanisms, such as the expression of fibrosis-related genes (e.g., TGF-β, α-SMA), limits the understanding of why MO failed to exhibit antifibrotic effects in this model. Future studies should address these aspects to better elucidate MO’s mechanisms. We selected two doses of MO based on previous studies (Wang et al., 2010), but exploring a broader range of dosing regimens could enhance the study’s insights. A novel aspect distinguishing our study is the acute administration of the tested compound immediately prior to the surgical procedure. In most prior studies, MO was administered for at least several days before the IR procedure, potentially allowing cellular saturation in hepatocytes, which we could not achieve with administration on the day before the procedure. This is the first study to assess the effects of acute MO administration, which likely accounts for the differences observed between our findings and those of prior studies. To better understand MO’s potential in hepatic IR injury, future studies should incorporate longer pre-procedure dosing regimens.

## Data Availability

The raw data supporting the conclusions of this article will be made available by the authors, without undue reservation.
